# Psychological Contributors to Purpose in Life among a Culturally Diverse Aging Cohort

**DOI:** 10.1002/alz.085309

**Published:** 2025-01-09

**Authors:** Uyen TM Vu, Sarah Tomaszewski Farias, Alyssa Weakley, Danielle J. Harvey, Oanh L. Meyer

**Affiliations:** ^1^ University of California, Davis School of Medicine, Sacramento, CA USA

## Abstract

**Background:**

Purpose in life (PIL) refers to individuals’ derivation of meaning from life experiences, possession of a sense of direction and intentionality, and striving towards goals. PIL is associated with many positive health outcomes and reduced risk of cognitive decline and dementia in older adults. Importantly, PIL is potentially modifiable through intervention to reduce risk of Alzheimer’s disease and related disorders. However, it is a complex construct likely influenced by psychological, lifestyle, and demographic factors. This study examines factors contributing to PIL, with the long‐range goal to inform the development of intervention approaches.

**Methods:**

This was a cross‐sectional study of participants from the University of California Davis Alzheimer’s Disease Research Center Longitudinal Cohort with a PIL score (N = 191). The mean age was 76.3 (SD = 7.2). Most were female (65%) and cognitively normal (85.3%). A substantial percentage were from underrepresented groups (45%). Independent variables of interest were grit, positive affect, emotional support, self‐efficacy, loneliness, and participation in physical and recreational activities. Covariates included age, sex, race, and education. Individual and joint linear regression models were run to examine associations.

**Results:**

All variables were significantly associated with PIL in bivariate models, but only positive affect was associated with higher PIL in the joint model including all independent variables and covariates (B = 0.478, 95% CI [0.362, 0.655], p < .001). Loneliness was associated with lower PIL (B = ‐0.18, 95% CI [‐0.384, ‐0.053], p = .01).

**Conclusions:**

Psychological, lifestyle, and demographic factors are important to consider in their association with PIL. Our findings showed that positive affect was associated with higher PIL and loneliness with lower PIL. These results have implications for future prevention and interventions to increase PIL in older adults, which may improve cognition and health. There are currently limited interventions that explicitly target PIL, and they are mostly in the cancer population. Conversely there are several evidence‐based approaches to increase positive affect that may also increase sense of PIL. Additionally, the association between loneliness and PIL suggests that increasing social connection may also bolster PIL.

